# Physical activity improves stress load, recovery, and academic performance-related parameters among university students: a longitudinal study on daily level

**DOI:** 10.1186/s12889-024-18082-z

**Published:** 2024-02-24

**Authors:** Monika Teuber, Daniel Leyhr, Gorden Sudeck

**Affiliations:** 1 Institute of Sports Science, Faculty of Economics and Social Sciences, University of Tübingen, Tübingen, Germany; 2 Methods Center, Faculty of Economics and Social Sciences, University of Tübingen, Tübingen, Germany; 3 Interfaculty Research Institute for Sports and Physical Activity, University of Tübingen, Tübingen, Germany

**Keywords:** Physical activity, Physical activity breaks, Stress load, Recovery, Psychological detachment, Academic performance, Attention, Study ability, University students

## Abstract

**Background:**

Physical activity has been proven to be beneficial for physical and psychological health as well as for academic achievement. However, especially university students are insufficiently physically active because of difficulties in time management regarding study, work, and social demands. As they are at a crucial life stage, it is of interest how physical activity affects university students' stress load and recovery as well as their academic performance.

**Methods:**

Student´s behavior during home studying in times of COVID-19 was examined longitudinally on a daily basis during a ten-day study period (*N* = 57, aged *M* = 23.5 years, *SD* = 2.8, studying between the 1st to 13th semester (*M* = 5.8, *SD* = 4.1)). Two-level regression models were conducted to predict daily variations in stress load, recovery and perceived academic performance depending on leisure-time physical activity and short physical activity breaks during studying periods. Parameters of the individual home studying behavior were also taken into account as covariates.

**Results:**

While physical activity breaks only positively affect stress load (functional stress b = 0.032, *p* < 0.01) and perceived academic performance (b = 0.121, *p* < 0.001), leisure-time physical activity affects parameters of stress load (functional stress: b = 0.003, *p* < 0.001, dysfunctional stress: b = -0.002, *p* < 0.01), recovery experience (b = -0.003, *p* < 0.001) and perceived academic performance (b = 0.012, *p* < 0.001). Home study behavior regarding the number of breaks and longest stretch of time also shows associations with recovery experience and perceived academic performance.

**Conclusions:**

Study results confirm the importance of different physical activities for university students` stress load, recovery experience and perceived academic performance in home studying periods. Universities should promote physical activity to keep their students healthy and capable of performing well in academic study: On the one hand, they can offer opportunities to be physically active in leisure time. On the other hand, they can support physical activity breaks during the learning process and in the immediate location of study.

**Supplementary Information:**

The online version contains supplementary material available at 10.1186/s12889-024-18082-z.

## Introduction

Physical activity (PA) takes a particularly key position in health promotion and prevention. It reduces risks for several diseases, overweight, and all-cause mortality [1] and is beneficial for physical, psychological and social health [2–5] as well as for academic achievement [6, 7]. However, PA levels decrease from childhood through adolescence and into adulthood [8–10]. Especially university students are insufficiently physically active according to health-oriented PA guidelines [11] because of academic workloads as well as difficulties in time management regarding study, work, and social demands [12]. Due to their independence and increasing self-responsibility, university students are at a crucial life stage. In this essential and still educational stage of the students´ development, it is important to study their PA behavior. Furthermore, PA as health behavior represents one influencing factor which is considered in the analytical framework of the impact of health and health behaviors on educational outcomes which was developed by the authors Suhrcke and de Paz Nieves [13, 14]. In light of this, the present study examines how PA affects university students' academic situations.

Along with the promotion of PA, the reduction of sedentary behavior has also become a crucial part of modern health promotion and prevention strategies. Spending too much time sitting increases many health risks, including the risk of obesity [15], diabetes [16] and other chronic diseases [15], damage to muscular balances, bone metabolism and musculoskeletal system [17] and even early death [15]. University students are a population that has shown the greatest increase in sedentary behavior over the last two decades [18]. In Germany, they show the highest percentage of sitting time among all working professional groups [19]. Long times sitting in classes, self-study learning, and through smartphone use, all of which are connected to the university setting and its associated behaviors, might be the cause of this [20, 21]. This goes along with technological advances which allow students to study in the comfort of their own homes without changing locations [22].

To counter a sedentary lifestyle, PA is crucial. In addition to its physical health advantages, PA is essential for coping with the intellectual and stress-related demands of academic life. PA shows positive associations with stress load and academic performance. It is positively associated with learning and educational success [6] and even shows stress-regulatory potential [23]. In contrast, sedentary behavior is associated with lower cognitive performance [24]. Moreover, theoretical derivations show that too much sitting could have a negative impact on brain health and diminish the positive effects of PA [16]. Given the theoretical background of the stressor detachment model [25] and the cybernetic approach to stress management in the workplace [26], PA can promote recovery experience, it can enhance academic performance, and it is a way to reduce the impact of study-related stressors on strain. Load-related stress response can be bilateral: On the one hand, it can be functional if it is beneficial to help cope with the study demands. On the other hand, it can be dysfunctional if it puts a strain on personal resources and can lead to load-related states of strain [27]. Thus, both, the promotion of PA and reduction of sedentary behavior are important for stress load, recovery, and performance in student life, which can be of particular importance for students in an academic context.

A simple but (presumably) effective way to integrate PA and reduce sedentary behavior in student life are short PA breaks. Due to the exercises' simplicity and short duration, students can perform them wherever they are — together in a lecture or alone at home. Short PA breaks could prevent an accumulation of negative stressors during the day and can help with prolonged sitting as well as inactivity. Especially in the university setting, evidence of the positive effects of PA breaks exists for self-perceived physical and psychological well-being of the university students [28]. PA breaks buffer university students’ perceived stress [29] and show positive impacts on recovery need [30] and better mood ratings [31, 32]. In addition, there is evidence for reduction in tension [30], overall muscular discomfort [33], daytime sleepiness or fatigue [33, 34] and increase in vigor [34] and experienced energy [30]. This is in line with cognitive, affective, behavioral, and biological effects of PA, all categorized as palliative-regenerative coping strategies, which addresses the consequences of stress-generating appraisal processes aiming to alleviate these consequences (palliative) or restore the baseline of the relevant reaction parameter (regenerative) [35, 36]. This is achieved by, for example, reducing stress-induced cortisol release or tension through physical activity (reaction reduction) [35]. Such mechanisms are also in accordance with the previously mentioned stressor detachment model [25]. Lastly, there is a health-strengthening effect that impacts the entire stress-coping-health process, relying on the compensatory effects of PA which is in accordance to the stress-buffering effect of exercise [37]. Health, in turn, effects educational outcomes [13, 14]. Therefore, stress regulating effects are also accompanied with the before mentioned analytical framework of the impact of health and health behaviors on educational outcomes [13, 14].

**Fig. 1:**
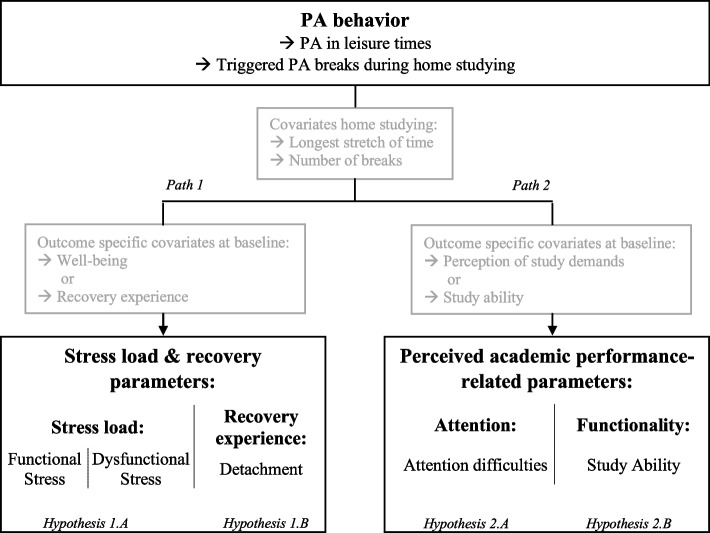
Overview of the assumed effects and investigated hypotheses of physical activity (PA) behavior on variables of stress load and recovery and perceived academic performance-related parameters

Focusing on the effects of PA, this study is guided by an inquiry into how PA affects university students' stress load and recovery as well as their perceived academic performance. For that reason, the student´s behavior during home studying in times of COVID-19 is examined, a time in which reinforced prolonged sitting, inactivity, and a negative stress load response was at a high [38–42]. Looking separately on the relation of PA with different parameters based on the mentioned evidence, we assume that PA has a positive impact on stress load, recovery, and perceived academic performance-related parameters. Furthermore, a side effect of the home study behavior on the mentioned parameters is assumed regarding the accumulation of negative stressors during home studying. These associations are presented in Fig. 1 and summarized in the following hypotheses:

- Hypothesis 1 (path 1): Given that stress load always occurs as a duality—beneficial if it is functional for coping, or exhausting if it puts a strain on personal resources [27] – we consider two variables for stress load: functional stress and dysfunctional stress. In order to reduce the length of the daily surveys, we focused the measure of recovery only on the most obvious and accessible component of recovery experience, namely psychological detachment. PA (whether performed in leisure-time or during PA breaks) encourages functional stress and reduce dysfunctional stress (1.A) and has a positive effect on recovery experience through psychological detachment (1.B).
- Hypothesis 2 (path 2): The academic performance-related parameters attention difficulties and study ability are positively influenced by PA (whether done in leisure-time or during PA breaks). We have chosen to assess attention difficulties for a cognitive parameter because poor control over the stream of occurring stimuli have been associated with impairment in executive functions or academic failure [43–46]. Furthermore, we have assessed the study ability to refer to the self-perceived feeling of functionality regarding the demands of students. PA reduces self-reported attention difficulties (2.A) and improves perceived study ability, indicating that a student feels capable of performing well in academic study (2.B).
- Hypothesis 3: We assume that a longer time spent on studying at home (so called home studying) could result in higher accumulation of stressors throughout the day which could elicit immediate stress responses, while breaks in general could reduce the influence of work-related stressors on strain and well-being [47, 48]. Therefore, the following covariates are considered for secondary effects:
  - the daily longest stretch of time without a break spent on home studying
  - the daily number of breaks during home studying

## Methods

### Study setting

The study was carried out during the COVID-19 pandemic containment phase. It took place in the middle of the lecture period between 25th of November and 4th of December 2020. Student life was characterized by home studying and digital learning. A so called “digital semester” was in effect at the University of Tübingen when the study took place. Hence, courses were mainly taught online (e.g., live or via a recorded lecture). Other events and actions at the university were not permitted. As such, the university sports department closed in-person sports activities. For leisure time in general, there were contact restrictions (social distancing), the performance of sports activities in groups was not permitted, and sports facilities were closed.

Thus, the university sports department of the University of Tübingen launched various online sports courses and the student health management introduced an opportunity for a new digital form of PA breaks. This opportunity provided PA breaks via videos with guided physical exercises and health-promoting explanations for a PA break for everyday home studying: the so called “Bewegungssnack digital” [in English “exercise snack digital” (ESD)] [49]. The ESD videos took 5–7 min and were categorized into three thematic foci: activation, relaxation, and coordination. Exercises were demonstrated by one or two student exercise leaders, accompanied by textual descriptions of the relevant execution features of each exercise.

### Participants

Participants were recruited within the framework of an intervention study, which was conducted to investigate whether a digital nudging intervention has a beneficial effect on taking PA breaks during home study periods [49]. Students at the University of Tübingen which counts 27,532 enrolled students were approached for participation through a variety of digital means: via an email sent to those who registered for ESD course on the homepage of the university sports department and to all students via the university email distribution list; via advertisement on social media of the university sports department (Facebook, Instagram, YouTube, homepage). Five tablets, two smart watches, and one iPad were raffled off to participants who engaged actively during the full study period in an effort to motivate them to stick with it to the end. In any case, participants knew that the study was voluntary and that they would not suffer any personal disadvantages should they opt out. There was a written informed consent prompt together with a prompt for the approval of the data protection regulations immediately within the first questionnaire (T0) presented in a mandatory selection field. Positive ethical approval for the study was given by the first author´s institution´s ethics committee of the faculty of the University of Tübingen.

Participants (*N* = 57) who completed the daily surveys on at least half of the days of the study period, were included in the sample (male = 6, female = 47, diverse = 1, not stated = 3). As not all subjects provided data on all ten study days, the total number of observations was between 468 and 540, depending on the variable under study (see Table 1). The average number of observations per subject was around eight. Their age was between 18 and 32 years (*M* = 23.52, *SD* = 2.81) and they were studying between the 1st to 13th semester (*M* = 5.76, *SD* = 4.11) within the following major courses of study: mathematical-scientific majors (34.0%), social science majors (22.6%), philosophical majors (18.9%), medicine (13.2%), theology (5.7%), economics (3.8%), or law (1.9%). 20.4% of the students had on-site classroom teaching on university campus for at least one day a week despite the mandated digital semester, as there were exceptions for special forms of teaching.

**Table 1:** Descriptive statistics of the variables used in the analysis with *n* representing the total number of observations on a daily level (468 < *n* < 540) and on individual level (53 < *n* < 54)

	*Variables*	*M (SD)*	*n*
***Daily outcome variables***			
*Variables for hypothesis 1*	Functional stress (1.A)	3.55 (1.10)	499
	Dysfunctional stress (1.A)	2.47 (1.01)	499
	Psychological detachment (1.B)	3.24 (1.01)	506
*Variables for hypothesis 2*	Attention difficulties (2.A)	2.32 (0.75)	501
	SAI (2.B)	12.52 (3.20)	500
***Independent variables***			
*PA behavior*	PA break time via ESD participation time (in min per day)	5.29 (6.30)	503
	LTPA time (in min per day)	54.77 (57.96)	501
*Variables for hypothesis 3*	Number of breaks during homes studying	2.92 (2.26)	475
	Longest stretch of time without a break spent on home studying (in hours)	1.87 (1.16)	468
*Covariates at T0*	Age (in years)	23.52 (2.78)	54
	Well-being	11.43 (4.12)	53
	Detachement	2.37 (0.79)	54
	Study demands scale	3.49 (0.88)	54
	SAI	12.67 (3.12)	54

### Design and procedures

To examine these hypothesized associations, a longitudinal study design with daily surveys was chosen following the suggestion of the day-level study of Feuerhahn et al. (2014) and also of Sonnentag (2001) measuring recovery potential of (exercise) activities during leisure time [50, 51]. Considering that there are also differences between people at the beginning of the study period, initial base-line value variables respective to the outcomes measured before the study period were considered as independent covariates. Therefore, the well-being at baseline serves as a control for stress load (2.A), the psychological detachment at baseline serves as a control for daily psychological detachment (2.B), the perception of study demands serves as a control for self-reported attention difficulties (1.A), and the perceived study ability at baseline serves as a control for daily study ability (2.B).

Subjects were asked to continue with their normal home study routine and additionally perform ESD at any time in their daily routine. Data were collected one to two days before (T0) as well as daily during the ten-day study period (Wednesday to Friday). The daily surveys (t_1_-t_10_) were sent by email at 7 p.m. every evening. Each day, subjects were asked to answer questions about their home studying behavior, study related requirements, recovery experience from study tasks, attention, and PA, including ESD participation. The surveys were conducted online using the UNIPARK software and were recorded and analyzed anonymously.

### Measures and covariates

In total, five outcome variables, two independent variables, and seven covariates were included in different analyses: three variables were used for stress load and recovery parameters, two variables for academic performance-related parameters, two variables for PA behavior, two variables for study behavior, four variables for outcome specific baseline values and one variable for age.

### Outcome variables

#### Stress load & recovery parameters (hypothesis 1)

Stress load was included in the analysis with two variables: functional stress and dysfunctional stress. Followingly, a questionnaire containing a word list of adjectives for the recording of emotions and stress during work (called “Erfassung von Emotionen und Beanspruchung “ in German, also known as EEB [52]) was used. It is an instrument which were developed and validated in the context of occupational health promotion. The items are based on mental-workload research and the assessment of the stress potential of work organization [52]. Within the questionnaire, four mental and motivational stress items were combined to form a functional stress scale (energetic, willing to perform, attentive, focused) (α = 0.89) and four negative emotional and physical stress items were combined to form dysfunctional stress scale (nervous, physically tensioned, excited, physically unwell) (α = 0.71). Participants rated the items according to how they felt about home studying in general on the following scale (adjustment from “work” to “home studying”): hardly, somewhat, to some extent, fairly, strongly, very strongly, exceptionally.

Recovery experience was measured via psychological detachment. Therefore, the dimension “detachment” of the Recovery Experience Questionnaire (RECQ [53]) was adjusted to home studying. The introductory question was "How did you experience your free time (including short breaks between learning) during home studying today?". Students responded to four statements based on the extent to which they agreed or disagreed (not at all true, somewhat true, moderately true, mostly true, completely true). The statements covered subjects such as forgetting about studying, not thinking about studying, detachment from studying, and keeping a distance from student tasks. The four items were combined into a score for psychological detachment (α = 0.94).

#### Academic performance-related parameters (hypothesis 2)

Attention was assessed via the subscale “difficulty maintaining focused attention performance” of the “Attention and Performance Self-Assessment” (ASPA, AP-F2 [54]). It contains nine items with statements about disturbing situations regarding concentration (e.g. “Even a small noise from the environment could disturb me while reading.”). Participants had to answer how often such situations happened to them on a given day on the following scale: never, rarely, sometimes, often, always. The nine items were combined into the AP-F2 score (α = 0.87).

The perceived study ability was assessed using the study ability index (SAI [55]). The study ability index captures the current state of perceived functioning in studying. It is based on the Work Ability Index by Hasselhorn and Freude ([56]) and consists of an adjusted short scale of three adapted items in the context of studying. Firstly, (a) the perceived academic performance was asked after in comparison to the best study-related academic performance ever achieved (from 0 = completely unable to function to 10 = currently best functioning). Secondly, the other two items were aimed at assessing current study-related performance in relation to (b) study tasks that have to be mastered cognitively and (c) the psychological demands of studying. Both items were answered on a five-point Likert scale (1 = very poor, 2 = rather poor, 3 = moderate, 4 = rather good, 5 = very good). A sum index, the SAI, was formed which can indicate values between 2 and 20, with higher values corresponding to higher assessed functioning in studies (α = 0.86). In a previous study it already showed satisfying reliability (α = 0.72) [55].

### Independent variables

#### PA behavior

Two indicators for PA behavior were included via self-reports: the time spent on ESD and the time spent on leisure-time PA (LTPA). Participants were asked the following overarching question daily: “How much time did you spend on physical activity today and in what context”. For the independent variable time spent on PA breaks, participants could answer the option “I participated in the Bewegungssnack digital” with the amount of time they spent on it (in minutes). To assess the time spent on LTPA besides PA breaks, participants could report their time for four different contexts of PA which comprised two forms: Firstly, structured supervised exercise was reported via time spent on (a) university sports courses and (b) other organized sports activities. Secondly, self-organized PA was indicated via (c) independent PA at home, such as a workout or other physically demanding activity such as cleaning or tidying up, as well as via (d) independent PA outside, like walking, cycling, jogging, a workout or something similar. Referring to the different domains of health enhancing PA [57], the reported minutes of these four types of PA were summed up to a total LTPA value. The total LTPA value was included in the analysis as a metric variable in minutes.

#### Covariates (hypothesis 3)

Regarding hypothesis 3 and home study behavior, the longest daily stretch of time without a break spent on home studying (in hours) and the daily number of breaks during home studying was assessed. Therein, participants had to answer the overarching question “How much time did you spend on your home studying today?” and give responses to the items: (1) longest stretch of time for home studying (without a break), and (2) number of short and long breaks you took during home studying.

In principle, efforts were made to control for potential confounders at the individual level (level 2) either by including the baseline measure (T0) of the respective variable or by including variables assessing related trait-like characteristics for respective outcomes. The reason why related trait-like characteristics were used for the outcomes was because brief assessments were used for daily surveys that were not concurrently employed in the baseline assessment. To enable the continued use of controlling for person-specific baseline characteristics in the analysis of daily associations, trait-like characteristics available from the baseline assessment were utilized as the best possible approximation.To sum up, four outcome specific baseline value variables were measured before the study period (at T0). The psychological detachment with the RECQ (α = 0.87) [53] was assessed at the beginning to monitor daily psychological detachment. Further, the SAI [55] was assessed at the beginning of the study period to monitor daily study ability. To monitor daily stress load, which in part measures mental stress aspects and negative emotional stress aspects, the well-being was assessed at the beginning using the WHO-Five Well-being Index (WHO-5 [58]). It is a one-dimensional self-report measure with five items. The index value is the sum of all items, with higher values indicating better well-being. As the well-being and stress load tolerance may linked with each other, this variable was assumed to be a good fit with the daily stress load indicating mental and emotional stress aspects. With respect to student life, daily academic performance-related attention was monitored with an instrument for the perception of study demands and resources (termed “Berliner Anforderungen Ressourcen-Inventar – Studierende” in German, the so-called BARI-S [59]). It contains eight items which capture overwork in studies, time pressure during studies, and the incompatibility of studies and private life. All together they form the BARI-S demand scale (α = 0.85) which was included in the analysis. As overwork and time pressure may result in attention difficulties (e.g. Elfering et al., 2013), this variable was assumed to have a good fit with academic performance-related attention [60]. Additionally, age in years at T0 was considered as a sociodemographic factor.

### Statistical analysis

Since the study design provided ten measurement points for various people, the hierarchical structure of the nested data called for two-level analyses. Pre-analyses of Random-Intercept-Only models for each of the outcome variables (hypothesis 1 to 3) revealed an Intra-Class-Correlation (*ICC*) of at least 0.10 (range 0.26 – 0.64) and confirmed the necessity to perform multilevel analyses [61]. Specifically, the day-level variables belong to Level 1 (ESD time, LTPA time, longest stretch of time without a break spent on home studying, daily number of breaks during home studying). To analyze day-specific effects within the person, these variables were centered on the person mean (cw = centered within) [50, 62–64]. This means that the analyses’ findings are based on a person’s deviations from their average values. The variables assessed at T0 belong to Level 2, which describe the person level (psychological detachment baseline, SAI baseline, well-being, study demands scale, age). These covariates on person level were centered around the grand mean [50] indicating that the analyses’ findings are based how far an individual deviates from the sample's mean values. As a result, the models’ intercept reflects the outcome value of an average student in the sample at his/her daily average behavior in PA and home study when all parameters are zero. For descriptive statistics SPSS 28.0.1.1 (IBM) and for inferential statistics R (version 4.1.2) were used. The hierarchical models were calculated using the package lme4 with the lmer-function in R in the following steps [65]. The Null Model was analyzed for all models first, with the corresponding intercept as the only predictor. Afterwards, all variables were entered. The regression coefficient estimates (”b”) were considered for statistical significance for the models and the respective BIC was provided.

In total, five regression models with ‘PA break time’ and ‘LTPA time’ as independent variables were computed due to the five measured outcomes of the present study. Three models belonged to hypothesis 1 and two models to hypothesis 2.

- Hypothesis 1: To test hypothesis 1.A two outcome variables were chosen for two separate models: ‘functional stress’ and ‘dysfunctional stress’. Besides the PA behavior variables, the ‘number of breaks’, the ‘longest stretch of time without a break spent on home studying’, ‘age’, and the ‘well-being’ at the beginning of the study as corresponding baseline variable to the output variable were also included as independent variables in both models. The outcome variable ‘psychological detachment’ was utilized in conjunction with the aforementioned independent variables to test hypotheses 1.B, with one exception: psychological detachment at the start of the study was chosen as the corresponding baseline variable.
- Hypothesis 2: To investigate hypothesis 2.A the outcome variable ‘attention difficulties’ was selected. Hypothesis 2.B was tested with the outcome variables ‘study ability’. Both models included both PA behavior variables as well as the ‘number of breaks’, the ‘longest stretch of time without a break spent on home studying’, ‘age’ and one corresponding baseline variable each: the ‘study demand scale’ at the start of the study for ‘attention difficulties’ and the ‘SAI’ at the beginning of the study for the daily ‘study ability’.
- Hypothesis 3: In addition to both PA behavior variables, age and one baseline variable that matched the outcome variable, the covariates ‘daily longest stretch of time spent on home studying’ and ‘daily number of breaks during home studying’ were included in the models for all five outcome variables.

### Handling missing data

The dataset had up to 18% missing values (most exhibit the variables ‘daily longest stretch of time without a break spent on home studying’ with 17.89% followed by ‘daily number of breaks during homes studying’ with 16.67%, and ‘functional / dysfunctional stress’ with 12.45%). Therefore, a sensitivity analysis was performed using the multiple imputation mice-package in the statistical program R [66], the package howManyImputation based on Von Hippel (2020, [67]), and the additional broom package [68]. The results of the models remained the same, with one exception for the Attention Difficulties Model: The daily longest stretch of time without a break spent on home studying showed a significant association (Table 1 in supplement). Due to this almost perfect consistency of results between analyses based on the dataset with missing data and those with imputed data alongside the lack of information provided by the packages for imputed datasets, we decided to stick with the main analysis including the missing data. Thus, in the following the results of the main analysis without imputations are presented.

## Results

Table 1 shows the descriptive statistics of the variables used in the analysis. An overview of the analysed models is presented in Table 2.

**Table 2:** Overview of the models

			**Hypothesis 1: Stress load & recovery parameters** **Estimate b (*SE*)**			**Hypothesis 2: Academic performance-related parameters** **Estimate b (*SE*)**	
			**Functional Stress Model**	**Dysfunctional Stress Model**	**Detachment Model**	**Attention Difficulties Model**	**Study Ability Model**
	*Intercept*		3.594*** **(0.102)**	2.417*** **(0.114)**	3.227*** **(0.081)**	2.315*** **(0.067)**	12.583*** **(0.236)**
*Level 1* time-varying	*PA behavior*	PA breaks via ESD min (cw2)	0.032** **(0.010)**	-0.000 (0.008)	0.010 (0.010)	-0.002 (0.007)	0.121*** **(0.033)**
		LTPA min (cw2)	0.003*** **(0.001)**	-0.002** **(0.001)**	0.003*** **(0.001)**	-0.003*** **(0.001)**	0.012*** **(0.003)**
	*Covariates*	Number of breaks (cw2)	0.031 (0.025)	-0.033 (0.020)	-0.058** **(0.022)**	-0.000 (0.015)	0.183* **(0.074)**
		Longest stretch of time without a break spent on home studying (cw2)	0.004 (0.043)	-0.027 (0.034)	-0.120** **(0.042)**	0.040 (0.028)	0.253 (0.139)
*Level 2 *time invariant		Age (c2)	0.032 (0.037)	0.005 (0.041)	0.013 (0.030)	0.023 (0.024)	0.044 (0.086)
	*Outcome specific baseline covariates *^*a)*^	T0 well-being (c2)	0.089*** **(0.025)**	-0.035 (0.028)			
		T0 detachment (c2)			0.471*** **(0.103)**		
		T0 study demands scale (c2)				0.240** **(0.076)**	
		T0 SAI (c2)					0.335*** **(0.075)**
Marginal *R*^*2*^ / Conditional *R*^*2*^			0.133 / 0.530	0.027 / 0.647	0.180 / 0.457	0.126 / 0.514	0.175 / 0.393
*BIC*			1120.7	966.1	1113.9	802.7	2148.2

*Cw* Centered within, *c2* Grand centered, *PA* Physical activity, *ESD* Exercise snack digital, *LTPA* Leisure-times physical activity, *SE* Standard error

^a^Those variables were only included in the appropriate model since they are outcome-specific covariates

(* *p* < 0.05, ** *p* < 0.01. ***p < 0.001)

### Effects on stress load and recovery (hypothesis 1)

Hypothesis 1.A: The Model Functional Stress explained 13% of the variance by fixed factors (marginal *R*^*2*^ = 0.13), and 52% by both fixed and random factors (conditional *R*^*2*^ = 0.52). The time spent on ESD as well as the time spent on PA in leisure showed a positive significant influence on functional stress (b = 0.032, *p* < 0.01). The same applied to LTPA (b = 0.003, *p* < 0.001). The Model Dysfunctional Stress (marginal *R*^*2*^ = 0.027, conditional *R*^*2*^ = 0.647) showed only one significant result. The dysfunctional stress was only significantly negatively influenced by the time spent on LTPA (b = 0.002, *p* < 0.01).

Hypothesis 1.B: With the Model Detachment, fixed factors contributed 18% of the explained variance and fixed and random factors 46% of the explained variance for psychological detachment. Only the amount of time spent on LTPA revealed a positive impact on psychological detachment (b = 0.003, *p* < 0.001).

### Effects on academic performance-related parameters (hypothesis 2)

Hypothesis 2.A: The Model Attention Difficulties showed 13% of the variance explained by fixed factors, and 51% explained by both fixed and random factors. It showed a significant negative association only for the time spent on LTPA (b = 0.003, *p* < 0.001).

Hypothesis 2.B: The Model SAI showed 18% of the variance explained by fixed factors, and 39% explained by both fixed and random factors. There were significant positive associations for time spent on ESD (b = 0.121, *p* < 0.001) and time spent on LTPA (b = 0.012, *p* < 0.001). The same applied to LTPA (b = 0.012, *p* < 0.001).

### Effects of home study behavior (hypothesis 3)

Regarding the independent covariates for the outcome variables functional and dysfunctional stress, there were no significant results for the number of breaks during homes studying or the longest stretch of time without a break spent on home studying. Considering the outcome variable ‘psychological detachment’, there were significant results with negative impact for both study behavior variables: breaks during home studying (b = 0.058, *p* < 0.01) and daily longest stretch of time without a break (b = 0.120, *p* < 0.01). Evaluating the outcome variables ‘attention difficulties’, there were no significant results for the number of breaks during home studying or the longest stretch of time without a break spent on home studying. Testing the independent study behavior variables for the SAI, it increased with increasing number in daily breaks during homes studying relative to the person´s mean (b = 0.183, *p* < 0.05). No significant effect was found for the longest stretch of time without a break spent on home studying (*p* = 0.07).

The baseline covariates of the models showed expected associations and thus confirmed their inclusion. The baseline variables well-being showed a significant impact on functional stress (b = 0.089, *p* < 0.001), psychological detachment showed a positive effect on the daily output variables psychological detachment (b = 0.471, *p* < 0.001), study demand scale showed a positive association on difficulties in attention (b = 0.240, *p* < 0.01), and baseline SAI had a positive effect on the daily SAI (b = 0.335, *p* < 0.001).

## Discussion

The present study theorized that PA breaks and LTPA positively influence the academic situation of university students. Therefore, impact on stress load (‘functional stress’ and ‘dysfunctional stress’) and ‘psychological detachment’ as well as academic performance-related parameters ‘self-reported attention difficulties’ and ‘perceived study ability’ was taken into account. The first and second hypotheses assumed that both PA breaks and LTPA are positively associated with the aforementioned parameters and were confirmed for LTPA for all parameters and for PA breaks for functional stress and perceived study ability. The third hypothesis assumed that home study behavior regarding the daily number of breaks during home studying and longest stretch of time without a break spent on home studying has side effects. Detected negative effects for both covariates on psychological detachment and positive effects for the daily number of breaks on perceived study ability were partly unexpected in their direction. These results emphasize the key position of PA in the context of modern health promotion especially for students in an academic context.

Regarding hypothesis 1 and the detected positive associations for stress load and recovery parameters with PA, the results are in accordance with the stress-regulatory potential of PA from the state of research [23]. For hypothesis 1.A, there is a positive influence of PA breaks and LTPA on functional stress and a negative influence of LTPA on dysfunctional stress. Given the bilateral role of stress load, the results indicate that PA breaks and LTPA are beneficial for coping with study demands, and may help to promote feelings of joy, pride, and learning progress [27]. This is in line with previous evidence that PA breaks in lectures can buffer university students’ perceived stress [29], lead to better mood ratings [29, 31], and increase in motivation [28, 69], vigor [34], energy [30], and self-perceived physical and psychological well-being [28]. Looking at dysfunctional stress, the result point that LTPA counteract load-related states of strain such as inner tension, irritability and nervous restlessness or feelings of boredom [27]. In contrast, short PA breaks during the day could not have enough impact in countering dysfunctional stress at the end of the day regarding the accumulation of negative stressors during home studying which might have occurred after the participant took PA breaks. Other studies have been able to show a reduction in tension [30] and general muscular discomfort [33] after PA breaks. However, this was measured as an immediate effect of PA breaks and not with general evening surveys. Blasche and colleagues [34] measured effects immediately and 20 min after different kind of breaks and found that PA breaks led to an additional short‐ and medium‐term increase in vigor while the relaxation break lead to an additional medium‐term decrease in fatigue compared to an unstructured open break. This is consistent with the results of the present study that an effect of PA breaks is only observed for functional stress and not for dysfunctional stress. Furthermore, there is evidence that long sitting during lectures leads to increased fatigue and lower concentration [31, 70], which could be counteracted by PA breaks. For both types of stress loads, functional and dysfunctional stress, there is an influence of students´ well-being in this study. This shows that the stress load is affected by the way students have mentally felt over the last two weeks. The relevance of monitoring this seems important especially in the time of COVID-19 as, for example, 65.3% of the students of a cross-sectional online survey at an Australian university reported low to very low well-being during that time [71]. However, since PA and well-being can support functional stress load, they should be of the highest priority—not only as regards the pandemic, but also in general.

Looking at hypothesis 1.B; while there is a positive influence of LTPA on experienced psychological detachment, no significant influence for PA breaks was detected. The fact that only LTPA has a positive effect can be explained by the voluntary character of the activity [50]. The voluntary character ensures that stressors no longer affect the student and, thus, recovery as detachment can take place. Home studying is not present in leisure times, and thus detachment from study is easier. The PA break videos, on the other hand, were shot in a university setting, which would have made it more difficult to detach from study. In order to further understand how PA breaks affect recovery and whether there is a distinction between PA breaks and LTPA, future research should also consider other types of recovery (e.g. relaxation, mastery, and control). Additionally, different types of PA breaks, such as group PA breaks taken on-site versus video-based PA breaks, should be taken into account.

Considering the confirmed positive associations for academic performance-related parameters of hypothesis 2, the results are in accordance with the evidence of positive associations between PA and learning and educational success [6], as well as between PA breaks and better cognitive functioning [28]. Looking at the self-reported attention difficulties of hypothesis 2.A, only LTPA can counteract it. PA breaks showed no effects, contrary to the results of a study of Löffler and collegues (2011, [31]), in which acute effects of PA breaks could be found for higher attention and cognitive performance. Furthermore, the perception of study demands before the study periods has a positive impact on difficulties in attention. That means that overload in studies, time pressure during studies, and incompatibility of studies and private life leads to higher difficulties with attention in home studying. In these conditions, PA breaks might have been seen as interfering, resulting in the expected beneficial effects of exercise on attention and task-related participation behavior [72, 73] therefore remaining undetected. With respect to the COVID-19 pandemic, accompanying education changes, and an increase in student´s worries [74, 75], the perception of study demands could be affected. This suggests that especially in times of constraint and changes, it is important to promote PA in order to counteract attention difficulties. This also applies to post-pandemic phase.

Regarding the perceived academic performance of hypothesis 2.B, both PA breaks and LTPA have a positive effect on perceived study ability. This result confirms the positive short-term effects on cognition tasks [76]. It is also in line with the positive function of PA breaks in interrupting sedentary behavior and therefore counteracting the negative association between sitting behavior and lower cognitive performance [24]. Additionally, this result also fits with the previously mentioned positive relationship between LTPA and functional stress and between PA breaks and functional stress.

According to hypothesis 3, in relation to the mentioned stress load and recovery parameters, there are negative effects of the daily number of breaks during home studying and the longest stretch of time without a break spent on home studying on psychological detachment. As stressors result in negative activation, which impede psychological detachment from study during non-studying time [25], it was expected and confirmed that the longest stretch of time without a break spent on home studying has a negative effect on detachment. Initially unexpected, the number of breaks has a negative influence on psychological detachment, as breaks could prevent the accumulation of strain reactions. However, if the breaks had no recovery effect through successful detachment, the number might not have any influence on recovery via detachment. This is indicated by the PA breaks, which had no impact on psychological detachment. Since there are other ways to recover from stress besides psychological detachment, such as relaxation, mastery, and control [53], PA breaks must have had an additional impact in relation to the positive results for functional stress.

In relation to the mentioned academic performance-related parameters, only the number of breaks has a positive influence on the perceived study ability. This indicates that not only PA breaks but also breaks in general lead to better perceived functionality in studying. Paulus and colleagues (2021) found out that an increase in cognitive skills is not only attributed to PA breaks and standing breaks, but also to open breaks with no special instructions [28]. Either way, they found better improvement in self-perceived physical and psychological well-being of the university students with PA breaks than with open breaks. This is also reflected in the present study with the aforementioned positive effects of PA breaks on functional stress, which does not apply to the number of breaks.

Overall, it must be considered that the there is a more complex network of associations between the examined parameters. The hypothesized separate relation of PA with different parameters do not consider associations between parameters of stress load / recovery and academic performance although there might be a interdependency. Furthermore, moderation aspects were not examined. For example, PA could be a moderator which buffer negative effects of stress on the study ability [55]. Moreover, perceived study ability might moderate stress levels and academic performance. Further studies should try to approach and understand the different relationships between the parameters in its complexity.

### Limitations

Certain limitations must be taken into account. Regarding the imbalanced design toward more female students in the sample (47 female versus 6 male), possible sampling bias cannot be excluded. Gender research on students' emotional states during COVID-19, when this study took place, or students´ acceptance of PA breaks is diverse and only partially supplied with inconsistent findings. For example, during the COVID-19 pandemic, some studies reported that female students were associated with lower well-being [71] or worse mental health trajectories [75, 77]. Another study with a large sample of students from 62 countries reported that male students were more strongly affected by the pandemic because they were significantly less satisfied with their academic life [74]. However, Keating and colleges (2020) discovered that, despite the COVID-19 pandemic, females rated some aspects of PA breaks during lectures more positively than male students did. However, this was also based on a female slanted sample [78]. Further studies are needed to get more insights into gender bias.

Furthermore, the small sample size combined with up to 16% missing values comprises a significant short-coming. There were a lot of possibilities which could cause such missing data, like refused, forgotten or missed participation, technical problems, or deviation of the personal code for the questionnaire between survey times. Although the effects could be excluded by sensitive analysis due to missing data, the sample is still small. To generalize the findings, future replication studies are needed.

Additionally, PA breaks were only captured through participation in the ESD, the specially instructed PA break via video. Effects of other short PA breaks were not include in the study. However, participants were called to participate in ESD whenever possible, so the likelihood that they did take part in PA breaks in addition to the ESD could be ignored.

With respect to the baseline variables, it must be considered that two variables (stress load, attention difficulties) were adjusted not with their identical variable in T0, but with other conceptually associated variables (well-being index, BARI-S). Indeed, contrary to the assumption the well-being index does only show an association with functional stress, indicating that it does not control dysfunctional stress. Although the other three assumed associations were confirmed there might be a discrepancy between the daily measured variables and the variables measured in T0. Further studies should either proof the association between these used variables or measure the same variables in T0 for control the daily value of these variables.

Moreover, the measuring instruments comprised the self-assessed perception of the students and thus do not provide an objective information. This must be considered, especially for measuring cognitive and academic-performance-related measures. Here, existing objective tests, such as multiple choice exams after a video-taped lecture [72] might have also been used. Nevertheless, such methods were mostly used in a lab setting and do not reflect reality. Due to economic reasons and the natural learning environment, such procedures were not applied in this study. However, the circumstances of COVID-19 pandemic allowed a kind of lab setting in real life, as there were a lot of restrictions in daily life which limited the influence of other covariates. The study design provides a real natural home studying environment, producing results that are applicable to the healthy way that students learn in the real world. As this study took place under the conditions of COVID-19, new transformations in studying were also taken into account, as home studying and digital learning are increasingly part of everyday study.

However, the restrictions during the COVID-19 pandemic could result in a greater extent of leisure time per se. As the available leisure time in general was not measured on daily level, it is not possible to distinguish if the examined effects on the outcomes are purely attributable to PA. It is possible that being more physical active is the result of having a greater extent of leisure time and not that PA but the leisure time itself effected the examined outcomes. To address this issue in future studies, it is necessary to measure the proportion of PA in relation to the leisure time available.

Furthermore, due to the retrospective nature of the daily assessments of the variables, there may be overstated associations which must be taken into account. Anyway, the daily level of the study design provides advantages regarding the ability to observe changes in an individual's characteristics over the period of the study. This design made it possible to find out the necessity to analyze the hierarchical structure of the intraindividual data nested within the interindividual data. The performed multilevel analyses made it possible to reflect the outcome of an average student in the sample at his/her daily average behavior in PA and home study.

### Conclusion and practical implications

The current findings confirm the importance of PA for university students` stress load, recovery experience, and academic performance-related parameters in home studying. Briefly summarized, it can be concluded that PA breaks positively affect stress load and perceived study ability. LTPA has a positive impact on stress load, recovery experience, and academic performance-related parameters regarding attention difficulties and perceived study ability. Following these results, universities should promote PA in both fashions in order to keep their students healthy and functioning: On the one hand, they should offer opportunities to be physically active in leisure time. This includes time, environment, and structural aspects. The university sport department, which offers sport courses and provides sport facilities on university campuses for students´ leisure time, is one good example. On the other hand, they should support PA breaks during the learning process and in the immediate location of study. This includes, for example, providing instructor videos for PA breaks to use while home studying, and furthermore having instructors to lead in-person PA breaks in on-site learning settings like universities´ libraries or even lectures and seminars. This not only promotes PA, but also reduces sedentary behavior and thereby reduces many other health risks. Further research should focus not only on the effect of PA behavior but also of sedentary behavior as well as the amount of leisure time per se. They should also try to implement objective measures for example on academic performance parameters and investigate different effect directions and possible moderation effects to get a deeper understanding of the complex network of associations in which PA plays a crucial role.

### Supplementary Information

**Supplementary file 1.**

**Supplementary file 2.**

## Data Availability

The datasets used and analyzed during the current study are available from the corresponding author on reasonable request.

## Abbreviations

ASPA: Attention and Performance Self-Assessment
BARI-S: "Berliner Anforderungen Ressourcen-Inventar – Studierende" (instrument for the perception of study demands and resources)
Cw: Centered within
c2: Grand centered
EEB: “Erfassung von Emotionen und Beanspruchung “ (questionnaire containing a word list of adjectives for the recording of emotions and stress during work)
ESD: Exercise snack digital (special physical activity break offer)
*ICC*: Intra-Class-Correlation
LTPA: Leisure time physical activity
PA: Physical activity
RECQ: Recovery Experience Questionnaire
SAI: Study ability index
WHO-5: World Health Organization-Five Well-being index
