# Supplementary material for: Physical activity improves stress load, recovery, and academic performance-related parameters among university students: a longitudinal study on daily level
Source: BMC Public Health. 2024 Feb 24;24:598. doi: 10.1186/s12889-024-18082-z (PMC10893600; doi:10.1186/s12889-024-18082-z)
Supplement: Supplementary file 1 [file 12889_2024_18082_MOESM1_ESM.pdf]

Supplement

Supplement Table 1: Overview of the models tested with 46 imputed datasets (p < 0.05, ** p < 0.01, *** p < 0.001)*

			Stress load & recovery parameters<br>Estimate b (*SE*)			Academic performance-related parameters<br>Estimate b (*SE*)	
			**Functional Stress Model**	**Dysfunctional Stress Model**	**Detachment Model**	**Attention Difficulties Model**	**Study Ability Model**
	Inter-cept		**3.848***<br>(0.100)**	**2.464***<br>(0.104)**	**3.227***<br>(0.076)**	**2.292***<br>(0.063)**	**12.886***<br>(0.233)**
Level 1<br>time-varying	*PA behavior*	PA breaks vie ESD min (cw2)	**0.052***<br>(0.026)**	-0.005<br>(0.008)	0.009<br>(0.010)	-0.005<br>(0.007)	0.142***<br>(0.039)
Level 1<br>time-varying	*PA behavior*	LTPA min (cw2)	**0.004***<br>(0.001)**	**-0.002**<br>(0.001)**	**0.002**<br>(0.001)**	**-0.002***<br>(0.001)**	**0.011***<br>(0.003)**
Level 1<br>time-varying	*Covariates*	Number of breaks (cw2)	0.025<br>(0.037)	-0.020<br>(0.023)	**-0.060**<br>(0.021)**	-0.006<br>(0.017)	**0.178*<br>(0.079)**
Level 1<br>time-varying	*Covariates*	Longest stretch of time without a break spent on home studying (cw2)	-0.009<br>(0.079)	-0.023<br>(0.037)	**-0.126**<br>(0.040)**	0.049*<br>(0.028)	0.207<br>(0.151)
Level 2<br>time invariant	*Covariates*	Age (c2)	0.032<br>(0.036)	0.012<br>(0.043)	0.005<br>(0.026)	0.026<br>(0.021)	0.035<br>(0.089)
Level 2<br>time invariant	*Outcome specific baseline covariates* [a]	T0 well-being (c2)	**0.045*<br>(0.027)**	-0.027<br>(0.022)			
Level 2<br>time invariant	*Outcome specific baseline covariates* [a]	T0 detachment (c2)			**0.4730***<br>(0.102)**		
Level 2<br>time invariant	*Outcome specific baseline covariates* [a]	T0 study demands scale (c2)				**0.202***<br>(0.069)**	
Level 2<br>time invariant	*Outcome specific baseline covariates* [a]	T0 SAI (c2)					**0.248***<br>(0.080)**

[a] Those variables were only included in the appropriate model since they are outcome-specific covariates.

cw = centered within, c2 = grand centered, PA = physical activity, ESD = exercise snack digital, LTPA = leisure-times physical activity, SE = standard error
