# Supplementary material for: Physical activity improves stress load, recovery, and academic performance-related parameters among university students: a longitudinal study on daily level
Source: BMC Public Health. 2024 Feb 24;24:598. doi: 10.1186/s12889-024-18082-z (PMC10893600; doi:10.1186/s12889-024-18082-z)
Supplement: Supplementary file 2 [file 12889_2024_18082_MOESM2_ESM.pdf]

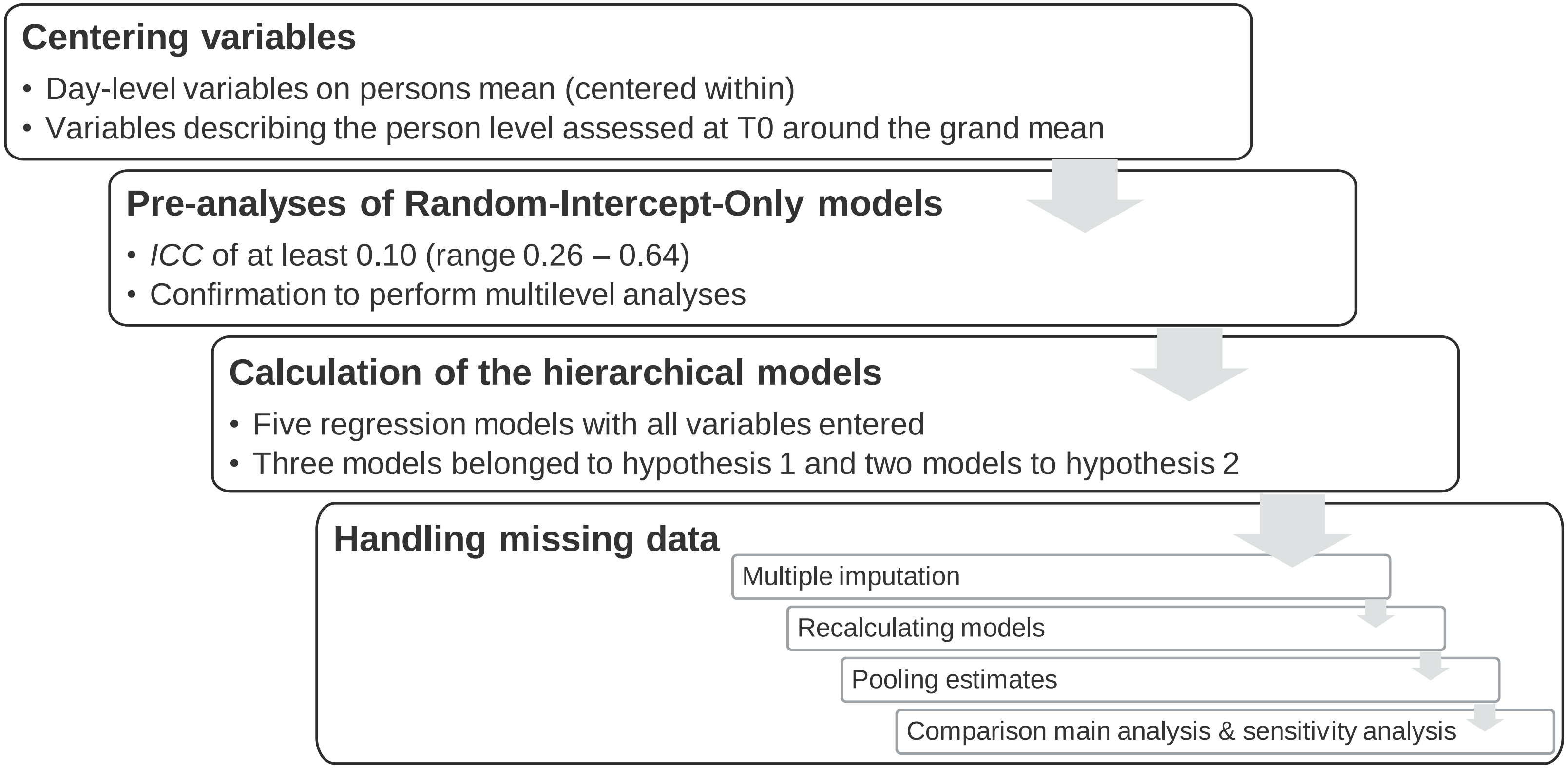
Centering variables
• Day-level variables on persons mean (centered within)
• Variables describing the person level assessed at T0 around the grand mean
Pre-analyses of Random-Intercept-Only models
• ICC of at least 0.10 (range 0.26 – 0.64)
• Confirmation to perform multilevel analyses
Calculation of the hierarchical models
• Five regression models with all variables entered
• Three models belonged to hypothesis 1 and two models to hypothesis 2
Handling missing data
Multiple imputation
Recalculating models
Pooling estimates
Comparison main analysis & sensitivity analysis
